# Supplementary material for: Resolving the full spectrum of human genome variation using Linked-Reads
Source: Genome Res. 2019 Apr;29(4):635–45. doi: 10.1101/gr.234443.118 (PMC6442396; doi:10.1101/gr.234443.118)
Supplement: Supplemental Material [file supp_gr.234443.118_Supplemental_longranger-2.2.zip › longranger-2.2.tar.bz2!/lr2.2-public/lib/stan/cmdstan/stan/doc/api/html/agrad_2rev_2matrix_2stan__print_8hpp_source.html]

Stan: src/stan/agrad/rev/matrix/stan\_print.hpp Source File


|  |  |
| --- | --- |
|  | Stan  2.5.0  probability, sampling & optimization |


- Main Page
- Related Pages
- Namespaces
- Classes
- Files

- File List
- File Members

All Classes Namespaces Files Functions Variables Typedefs Enumerations Enumerator Friends Macros Pages

- src
- stan
- agrad
- rev
- matrix

stan\_print.hpp

Go to the documentation of this file.

1 #ifndef STAN\_\_AGRAD\_\_REV\_\_MATRIX\_\_STAN\_PRINT\_HPP

2 #define STAN\_\_AGRAD\_\_REV\_\_MATRIX\_\_STAN\_PRINT\_HPP

3

4 #include <ostream>

5 #include <stan/agrad/rev/var.hpp>

6

7 namespace stan {

8  namespace agrad {

9

10  void stan\_print(std::ostream\* o, const var& x) {

11  \*o << x.val();

12  }

13

14  }

15 }

16 #endif

stan::agrad::stan\_print

void stan\_print(std::ostream \*o, const var &x)

**Definition:** stan\_print.hpp:10

stan::agrad::var::val

double val() const

Return the value of this variable.

**Definition:** var.hpp:209

var.hpp

stan::agrad::var

Independent (input) and dependent (output) variables for gradients.

**Definition:** var.hpp:27


---

[ Stan Home Page ]

*© 2011–2014,
Stan Development Team.*
